# A data-driven framework for biomarker discovery applied to optimizing modern clinical and preclinical trials on Alzheimer’s disease

**DOI:** 10.1093/braincomms/fcae438

**Published:** 2024-12-09

**Authors:** Isaac Llorente-Saguer, Neil P Oxtoby

**Affiliations:** UCL Hawkes Institute and Department of Medical Physics and Biomedical Engineering, University College London, London WC1E 6BT, UK; UCL Hawkes Institute and Department of Computer Science, University College London, London WC1E 6BT, UK

**Keywords:** Alzheimer, biomarker, imaging, tau, clinical trial

## Abstract

PET is used to measure tau protein accumulation in Alzheimer’s disease. Multiple biomarkers have been proposed to track disease progression, most notably the standardized uptake value ratio of PET tracer uptake in a target region of interest relative to a reference region, but literature suggests these region choices are nontrivial. This study presents and evaluates a novel framework, BioDisCVR, designed to facilitate the discovery of useful biomarkers, demonstrated on [^18^F]AV-1451 tau PET data in multiple cohorts. BioDisCVR enhances signal-to-noise by conducting a data-driven search through the space of possible combinations of regional tau PET signals into a ratio of two composite regions, driven by a user-defined fitness function. This study compares ratio-based biomarkers discovered by the framework with state-of-the-art standardized uptake value ratio biomarkers. Data used is tau PET regional measurements from 198 individuals from the Alzheimer’s Disease Neuroimaging Initiative database, used for discovery, and 42 from the Mayo Clinic Alzheimer’s Disease Research Center and Mayo Clinic Study of Aging (MCSA), used for external validation. Biomarkers are evaluated by calculating clinical trial sample size estimates for 80% power and 20% effect size. Secondary metrics are a measure of longitudinal consistency (standard deviation of linear mixed-effects model residuals), and separation between cognitive groups (*t*-statistic of the change over time due to being cognitively impaired). When applied to preclinical (secondary prevention with CU individuals) and clinical (treatment aimed at cognitively impaired individuals) trials on Alzheimer’s disease, our data-driven framework BioDisCVR discovered ratio-based tau PET biomarkers vastly superior to previous work, both reducing measurement error and sample size estimates for hypothetical clinical trials. Our analysis suggests remarkable potential for patient benefit (reduced exposure to health risks associated with experimental drugs) and substantial cost savings, through accelerated trials and reduced sample sizes. Our study supports the leveraging of data-driven methods like BioDisCVR for clinical benefit, with the potential to positively impact drug development in Alzheimer’s disease and beyond.

## Teaser

Llorente-Saguer and Oxtoby present a biomarker discovery framework, applied to tau Positron Emission Tomography data in Alzheimer’s disease. The resulting ratios of data-driven composite regions reduces sample size estimates by ∼80% in preclinical and clinical trials (externally validated), potentially accelerating drug development, reducing costs and enhancing patient safety.

## Introduction

Biomarkers have revolutionized medical research for everything from *in vivo* diagnosis (e.g. in Alzheimer’s disease) to detecting drug target engagement.^1^ This is particularly exciting in age-related neurodegenerative disease research, where an increasing number of disease-modifying treatments are emerging from clinical trials in Alzheimer’s disease.^2-4^ As these drugs reach healthcare settings, identifying optimal biomarkers will likely be key for supporting and informing patient management and treatment deployment decisions. However, no biomarker is perfect, which has ramifications for medical research and healthcare applications.

PET is a key technology in modern Alzheimer’s disease clinical trials for confirming clearance of pathology (amyloid and tau protein aggregates)^2-4^ and for trial eligibility.^4^ Both scenarios require a biomarker to be defined for the quantification of pathology. This has been the topic of numerous investigations in multiple PET modalities, using various statistical and biological approaches.

Ratio-based biomarkers (e.g. brain relative volumes or peptide ratios in blood or cerebrospinal fluid) have demonstrated great promise in diagnostic and screening applications.^5,6^ This is due in large part to the property of ratios cancelling out confounding factors—including biological factors, e.g. different blood flow rate, total brain volume or body mass, and methodological factors, e.g. instrumentation bias and repeatability, or batch effects. Indeed, the standard for positron emission tomography brain imaging in neurodegenerative disease is to quantify pathology using the standardized uptake value ratio (SUVR), which is the standardized uptake value (SUV) of a target region of interest with respect to the SUV of a reference region.

Previous data-driven investigations of optimal tau PET biomarkers for Alzheimer’s disease are mostly SUVR-based (note that the Centiloid^7^/CenTauR^8^ scales depend on SUVR). One prior study explored multiple image analysis pipelines and target/reference region choices in a search to optimize diagnostic separation and longitudinal precision of tau PET SUVR.^9^ Others sought to find a tau PET biomarker that would reduce clinical trial sample size estimates by combining known disease-affected regions in a weighted average,^10^ or proposing an individualized biomarker.^11^ Similar earlier work defined so-called ‘statistical’ regions of interest.^12-16^ All previous analyses are limited by the traditional SUVR mindset of using a reference region that is putatively both free of pathology and stable over time. However, the choice of a reference for SUVR in Alzheimer’s disease remains subjective and non-trivial.^17,18^ If common reference regions are interchangeable, their SUV values should be correlated. The fact that they are not^18^ hints at a fundamental problem with the current single-reference-region SUVR concept, which motivates further investigation such as the present study.

We turn the spotlight to a new question: is there a ratio of two composite regions, not restricted by the current SUVR paradigm, which is more useful in clinical applications? To be more specific, here we aim to find a data-driven tau PET biomarker for Alzheimer’s disease progression analyses. To achieve this, we developed a machine learning framework called BioDisCVR for discovering data-driven ratio-based biomarkers driven by a clinical objective.^19^ The framework is general (e.g. could be used on fluid biomarkers, or other features such as regional volumes, cortical thickness, cognitive scores) but here, we apply it to tau PET imaging data for clinical trials on Alzheimer’s disease.

## Materials and methods

Applied to regional tau PET SUV data, our framework finds a data-driven ratio-based biomarker with numerator and denominator each consisting of a data-driven composite region (combination of multiple sub-regions). We call the biomarker the composite value ratio (CVR) and name the biomarker discovery framework BioDisCVR (‘bio discover’). Our experiments are designed to discover CVRs that minimize the sample size estimate in modern randomized controlled clinical trials on Alzheimer’s disease. We compare the performance of CVR biomarkers against relevant benchmarks from the literature, demonstrating across multiple datasets.

### Study design

**Figure 1:**
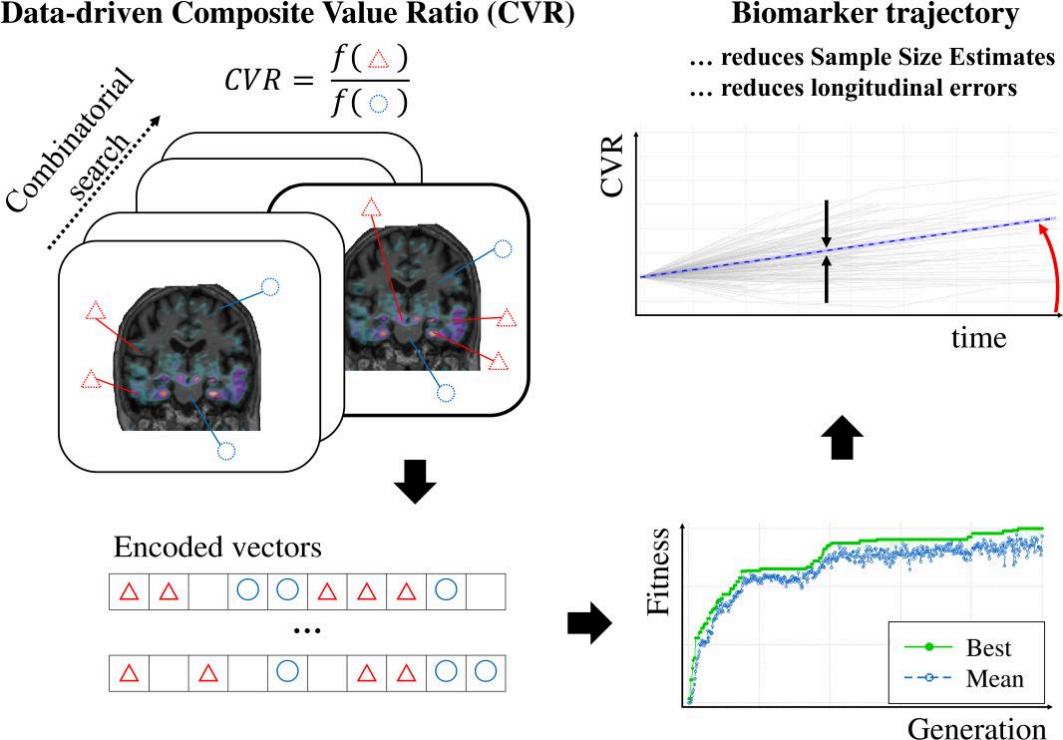
**Conceptual representation of the BioDisCVR framework.** A search algorithm is deployed to find a well-performing biomarker, defined as the ratio of signals f(triangles)/f(circles) in two composite brain regions (f(triangles) and f(circles)). The algorithm is driven by minimizing the sample size estimate needed for a hypothetical clinical trial, while maximizing the biomarker trajectory separation between cognitive groups (curved red arrow).

Figure 1 is a conceptual representation of the BioDisCVR framework. Our algorithm is described in BioDisCVR Framework and Benchmarks. Input features are regional brain volumes and regional PET SUV values (regions included are listed in Data). The algorithm searches through the combinatorial space (both numerator and denominator simultaneously) to find a ratio of composite regions driven by an application-specific fitness function. Here, we demonstrate BioDisCVR using two scenarios relevant to modern clinical trials on Alzheimer’s disease:

- Experiment 1: 54-month secondary prevention trial in cognitively unimpaired (CU) individuals at risk of Alzheimer’s disease (cf., the A4 Study^20^).
- Experiment 2: 18-month treatment trial in cognitively impaired (CI) individuals –cf., recent prominent trials of monoclonal antibodies Aducanumab (ENGAGE/EMERGE^2^), Lecanemab (Clarity-AD^3^) and Donanemab (TRAILBLAZER-ALZ 2^4^).
- Experiment 3: The same as Experiment 2, but with a subset of regions common to multiple datasets, to facilitate external validation. Here, we use the Alzheimer’s Disease Neuroimaging Initiative (ADNI) dataset for discovery and two datasets from the Mayo Clinic for validation (ADRC and MCSA). The datasets are described below in Data.

Our fitness function drives the algorithm to simultaneously reduce the sample size estimate (SSE) for a given clinical trial design, while maximizing cognitive group (biomarker) gradient separation (see Statistical Analysis). We report the SSE for 20% effect size and 80% power. Sample size estimate is an inverse square function of the effect size.

Extensive supplementary experiments are performed with multiple random initializations, alternative evaluation metrics, restricted search spaces (Supplementary Table 1) and regional ablation (Supplementary Fig. 1) to aid interpretability and comparability with the literature, while also demonstrating robustness of the BioDisCVR framework.

### Data

This study uses a discovery dataset to find and evaluate biomarkers, and an additional two cohorts for validation.

Discovery dataset: the ADNI. Data analysed are regional brain tau PET signal and MRI volumes from the ADNI database (downloaded on 23 February 2024 from adni.loni.usc.edu), using participants with available processed [^18^F]AV-1451 tau PET scans. Our inclusion criteria consists of being amyloid-positive at any visit (composite amyloid-PET SUVR above or equal to 0.78 for Florbetapir F18 and 0.74 for Florbetaben F18)^21^ and having at least two visits with concurrent tau-PET and T1w MRI scans. Although the linear mixed model we use needs three time-points per individual to return a measure of the residuals, we can (and do) include data from individuals having only two scans, as these data contribute to the intercept and gradient. Diagnosis is defined by the individual’s maximum level of cognitive impairment across available visits. Of the 391 participants having two or more eligible visits, 198 were amyloid-positive (48.7% females). Among these, 76 were CU, 122 CI, which is the aggregation of individuals with either mild cognitive impairment or dementia due to probable Alzheimer’s disease), of which 71 were labelled to have dementia due to probable Alzheimer’s disease. The PET scans were acquired across 48 different sites, with voxel resolutions of 6 and 8 mm. The majority of participants (about 94%) identify as belonging to a white ethnicity (see Discussion).

Validation datasets: Mayo Clinic Alzheimer's Disease Research Center (ADRC) and MCSA. Individuals with dementia due to aetiologies other than Alzheimer’s disease were discarded for this study (*N* = 5). The same criteria as with ADNI data were applied (three visits with AV-1451 tau PET, structural MRI scan and a measure of amyloid), with the exception of the amyloid cut-off: since the composite reference region is not available in these datasets, we used the available regions from the cortical summary region (that is made up of frontal, anterior/posterior cingulate, lateral parietal, lateral temporal regions: full list in Supplementary file Results.xlsx, tab ‘amyloid.target’)^21^ and ‘inferior cerebellum grey matter’ as the reference. Then, we fit a 2-component Gaussian mixture model^22^ (see Supplementary Fig. 2) and derived the cut-off of 1.53 for the ¹¹C-Pittsburgh Compound B PET, to determine amyloid positivity. This reduced the available individuals to 43 (1 CU, 13 MCI and 29 Ad). Because of the small sample size of the CU group, we only used the available 42 CI individuals (33.3% females).

Time between visits is shown for all experiments in Table 1. The majority of intervals are from 0.7 to 1.5 years, but some span 3 years. We did not use partial-volume-corrected SUVR data, as it has been shown to introduce errors.^9,23^

**Table 1:** Proportion of individuals per interval time between visits

Time between visits (years)	ADNI		Mayo
	CU	CI	CI
*t* ≤ 1.5	61%	72%	76%
1.5 > *t* ≤ 2.5	22%	17%	18%
2.5 > *t* ≤ 3.5	6%	6%	6%
*t* > 3.5	11%	5%	0%

Most individuals had visits separated by 0.7 to 1.5 years. Data from the ADNI and from the Mayo Clinic ADRC and MCSA, the last two grouped in the Mayo column.

BioDisCVR input features, as with classical machine learning models, can be the aggregation of any numerical variables. In our case, they consist of regional PET SUV and volumes for brain regions. In the Discovery dataset (ADNI), regions are defined by the Desikan-Killiany atlas,^24^ with the data provided on the Image & Data Archive run by the University of Southern California Laboratory of Neuro Imaging (LONI) for download in CSV format. For details of the processing pipelines, see ADNI’s manuscript^25^; this pipeline depends on a contemporaneous native space MRI that is segmented and parcellated with Freesurfer v7.1.1 (surfer.nmr.mgh.harvard.edu).^26^ For the Validation datasets, volume measures were obtained by the Mayo Clinic using the second version of the SUIT atlas.^27^ Off-target binding regions were excluded to ensure accurate quantification of tau pathology by minimizing interference from non-specific binding.^25^ The complete list of brain regions made available to our model is provided in the Supplementary File Results.xlsx (‘regions.available’ tab).

### BioDisCVR framework and benchmarks

In this study, we use our framework BioDisCVR to discover biomarkers tailored for a given clinical trial design. Here, we consider both preclinical and clinical trials (in CU/CI individuals, respectively) of fixed duration (see Study design).

#### BioDisCVR application to tau PET for randomized clinical trials

Given a set of regions, we encode the ratio of two composite regions in a vector of 0, 1 and 2 s, where 0 denotes numerator, 2 denotes denominator and 1 indicates regions not included in the ratio. To overcome the challenge of searching the intractably large space of permutations (over 10^40^, using the Stirling series approximation), we employ an objective-driven genetic algorithm^28^ consisting of tournament selection with 3 random picks, blend crossover, random adaptive mutation and a population of 32. The resulting ratio-based biomarker is called CVR and can consist of combinations of either SUV or SUVR because the reference region cancels out in the ratio:


(1) $$CVR=\;\frac{composite_{1}\;SUVR}{composite_{2}\;SUVR}=\frac{\frac{composite_{1}\;SUV}{reference\;SUV}}{\frac{composite_{2}\;SUV}{reference\;SUV}}=\frac{composite_{1}\;SUV}{composite_{2}\;SUV}$$

where $composite_{i}\;$ is the signal from any number of regions, combined in any way (e.g. volume-weighted, averaged).

To drive CVR towards minimizing the SSE of a hypothetical clinical trial while maintaining relevance to disease progression, we use a fitness/objective function equal to a measure of the trajectory separation between cognitive groups (see Statistical analysis below) divided by the square of the SSE. Here, the SSE is squared to slightly prioritize SSE over cognitive group separation, but this is a design choice for our experiments and not a requirement of the BioDisCVR framework. Adding group separation also forces the biomarker to increase in signal for the CI group with respect to the CU, facilitating interpretation and comparison with other biomarkers. We reduce the search space (again by choice, not a requirement) by using two biologically motivated priors: (i) no Braak1–3 regions^29^ in the denominator and (ii) no commonly used SUVR reference regions in the numerator.

#### Pipelines

Our experiments include several pipelines to investigate laterality (right–left hemisphere differences) and region size effects. Laterality is explored through averaged bilateral regions (indicated by the suffix-B in the biomarker nomenclature) versus left/right independent (indicated by the suffix-L in the biomarker nomenclature). Region size effects are explored through volume-weighted SUV versus simple mean SUV (mSUV). Supplementary Table 1 contains additional experiments where either the numerator or denominator is defined *a priori*.

#### Baselines

We compare the performance of CVR to SUVR biomarkers from the literature: meta-temporal^14^ (entorhinal cortex, parahippocampal gyrus, amygdala, inferior temporal gyrus, fusiform gyrus, middle temporal gyrus) divided by a composite reference region (eroded subcortical white matter, whole cerebellum and brainstem)^13^; highest tau-PET-positive data-driven stage (DDS,^11^ an adaptive individualized approach) divided by the inferior cerebellum grey matter. Additionally, we consider all combinations of composite target and reference regions we found in the literature: Braak stages,^29^ meta-temporal,^14^ mesial-temporal,^30^ temporoparietal,^30^ ‘rest’^30^ as target/numerator regions; and a set of popular reference regions (whole cerebellum, inferior cerebellar grey matter, composite reference region, eroded subcortical white matter, cerebellum cortex, brainstem, eroded subcortical white matter + inferior cerebellar grey matter, eroded subcortical white matter + whole cerebellum, whole cerebellum + brainstem, inferior cerebellar grey matter + brainstem, eroded subcortical white matter + inferior cerebellar grey matter + brainstem). Including composites calculated using both mean SUV and volume-weighted SUV, we compared our results with a total of 220 literature-inspired baseline biomarkers.

We report the best result found after 300 generations of the genetic algorithm for each of the constraints or scenarios. The top 5 biomarkers found, as well as the effect of different random initializations are shown in Supplementary File Results.xlsx, ‘Random.seeds_top.n’ tab. Our aim is not to show convergence or a focus on the search algorithm; rather, the evaluation of the biomarkers found by the algorithm. Thus, the 300 generations is arbitrary, although in 20 random initialization experiments with 500 generations each, 90% of the final performance was achieved within a mean of 140 generations (and below a standard deviation of 100), for both clinical and preclinical cases. Furthermore, the generations alone are not an indicator of the total number of combinations tested, as the genetic algorithm population size should also be taken into account.

### Statistical analysis

All analyses are performed in R^31^ (version 4.3.2). Our analysis involves calculating SSE, longitudinal group separation and longitudinal precision (defined below) for clinical trials on Alzheimer’s disease in either CU or CI participants. The SSE and group separation measures drive the genetic algorithm. Longitudinal group separation (*t*-statistic) and longitudinal precision (standard deviation of model residuals) of tau PET biomarkers are quantified using a linear mixed effects model^32^ fit to log(SUVR). Covariates sex, *APOE4* and education were not included because they did not significantly impact the intercept or the slope of the biomarker, replicating the analysis of Schwarz *et al.*^9^ Our linear mixed effects model includes correlated random slopes and intercepts, since this makes it invariant to time-shifts,^32^ and we use a relative time point (the average of all visits per individual). Furthermore, in the discovery cohort data (ADNI), a χ2 test indicated that the correlated random effects model fits the data better than the uncorrelated model, both for the CU ($\chi^{2}=13.38$, Df = 1, *P* < 0.001) and CI ($\chi^{2}=13.39$, Df = 1, *P* < 0.001). This is the formula used:


(2) $$\mathrm{SUV}R_{ij}=\beta_{0}+\beta_{1}\cdot {\mathrm{time}}_{ij}+\beta_{2}\cdot DX_{ij}+\beta_{3}\cdot {\mathrm{time}}_{ij}\cdot DX_{ij}+b_{0j}+b_{1j}{\mathrm{time}}_{ij}+\epsilon_{ij}$$

where:

- *i* indexes the observation,
- *j* indicates the individual,
- *DX* is a binary variable indicating whether the individual *j* belongs to the CI group (1) or not (0),
- *β*_0∼3_ are fixed effects,
- *b*_0*j*_ and *b*_1*j*_ are random effects (intercepts and slopes varying by individual),
- *ɛ_i_* is the residual error.

Cognitive group separation (CU versus CI) is quantified by the *t*-statistic between fixed effect gradients (*β*_3_ in Eq. 2). The measure of longitudinal repeatability is quantified using the standard deviation of the model residuals (*ɛ* in Eq. 2), which is a measure of the relative error with respect to the biomarker values. Finally, we use the longpower package^33^ to calculate the SSE for each of two hypothetical clinical trials designed for 80% power to reduce tau PET accumulation by 20% versus placebo: CU individuals, 54 months (Experiment 1, c.f. the A4 Study^20^); and CI individuals, 18 months (Experiment 2, c.f. ENGAGE/EMERGE,^2^ CLARITY-AD,^3^ TRAILBLAZER-ALZ 2^4^).

All metrics are provided with 95% confidence intervals, calculated using the model-based (semi-) parametric bootstrap for mixed models function from the lme4 package,^32^ version 1.1.35.1.

## Results

### Computation

Using a 12th Gen Intel(R) Core(TM) i7-12700H, with 2.70 GHz and 20 cores, we ran the search algorithm for <3 mins per experiment (Supplementary File Results.xlsx, tab Random.seeds_top.n, shows different random initializations of BioDisCVR).

### Experiment 1 (hypothetical 54-month preclinical AD trial)

**Figure 2:**
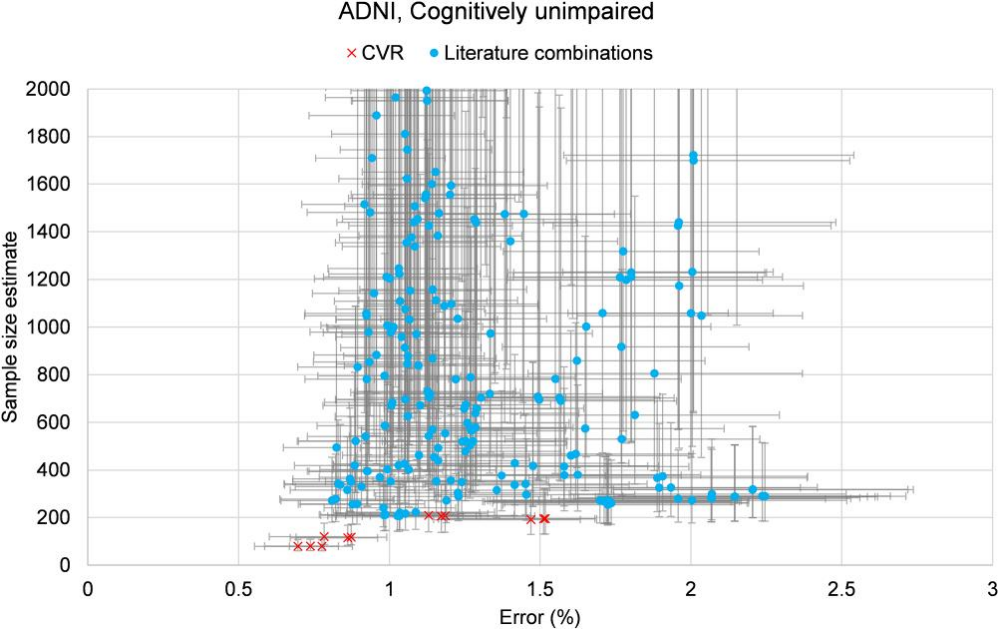
**Biomarker performance for Experiment 1.** The plot shows SSE against percentage error (longitudinal precision) for all biomarkers in Experiment 1, truncated at SSE ≤ 2000. CVR biomarkers (red crosses) showed superior performance to baseline comparators (blue dots), reducing both the SSE and the error. Error bars show 95% confidence intervals from bootstrapping. The SSE shown is the minimum number of individuals that would be needed per branch for a hypothetical preclinical trial (CU individuals), designed for 80% power and 20% effect size, for a duration of 4.5 years.

Figure 2 is a plot of SSE against percentage error (longitudinal precision) for all biomarkers in Experiment 1, truncated at SSE ≤ 2000. CVR biomarkers (red crosses) showed superior performance to baseline comparators (blue dots). Error bars show 95% confidence intervals from bootstrapping (see Statistical analysis).

Table 2 highlights selected biomarkers for Experiment 1 (all results are included in Supplementary material File Results.xlsx as a spreadsheet). Namely, specific literature biomarkers, plus the best-performing baseline (biomarker inspired by the literature) and our CVR biomarkers found by the algorithm. Overall, Table 2 shows that one of the CVR biomarkers always outperforms state-of-the-art biomarkers from the literature—for all metrics and, in particular, our primary goal of smaller SSE.

**Table 2:** Results of Experiment 1: biomarker performance for a 54-month clinical trial in an amyloid-positive, CU cohort

Biomarker	SSE	Separation	Repeatability
meta-temp/composite	626 (353, 1401)	4.37 (2.46, 6.49)	1.06 (0.82, 1.31)
DDS/inferior_cerebellum_gm	438 (259, 895)	3.48 (1.57, 5.7)	1.66 (1.32, 2.06)
(mSUV) mesial_temporal/ (ewm + cerebellum)	206 (143, 325)	0.17 (−1.84, 2.09)	1.03 (0.82, 1.3)
CVR-SUV-L	195 (131, 321)	4.2 (2.24, 6.25)	1.52 (1.19, 1.68)
**CVR-mSUV-L**	**80** (**61, 108)**	4.32 (2.31, 6.37)	**0.78** (**0.67, 0.88)**
CVR-SUV-B	205 (138, 338)	**4.43** (**2.61, 6.56)**	1.17 (0.91, 1.47)
CVR-mSUV-B	115 (84, 168)	3.3 (1.33, 5.43)	0.86 (0.67, 0.96)

Numbers in parentheses correspond to the 95% confidence interval. SSE is assessed with 80% power to detect a 20% effect size with respect to placebo, with two visits separated by 54 months. In bold, the best result per metric. Composite = average PET SUV of whole cerebellum, brainstem and eroded subcortical white matter. Inferior_cerebellum_gm = inferior cerebellum grey matter. ‘ewm + cerebellum’ indicate the average of the eroded subcortical white matter and the whole cerebellum. Our biomarkers have the prefix ‘CVR’, short for composite value ratio, with mSUV for mean SUV or SUV for volume-weighted SUV, and a suffix L or B indicating a lateral or bilateral regional analysis.

The best results for SSE were obtained with CVR-mSUV-L which is a ratio of simple-mean SUV combinations and laterality (separate hemispheric regions). The numerator included grey-matter regions from both hemispheres (superior frontal gyrus, amygdala), from the left hemisphere (caudal middle frontal gyrus, isthmus of cingulate gyrus, lingual gyrus, paracentral lobule, parahippocampal gyrus, postcentral gyrus, superior temporal gyrus), from the right hemisphere (lateral orbitofrontal gyrus). The denominator included grey- and white-matter regions from both hemispheres (precentral gyrus, brainstem, corpus callosum anterior, corpus callosum central, corpus callosum posterior), from the left hemisphere (insula, superior parietal lobule, ventral diencephalon), from the right hemisphere (lingual gyrus, paracentral lobule, postcentral gyrus, rostral anterior cingulate cortex).

The second-best SSE in Experiment 1 (about +45% SSE-worse) was obtained by CVR-mSUV-B, which combines bilateral mean SUV. The numerator included grey-matter regions from both hemispheres (entorhinal cortex, amygdala, inferior temporal gyrus, insula, medial orbitofrontal cortex, paracentral lobule, precuneus, superior frontal gyrus, temporal pole, transverse temporal gyrus). The denominator included grey- and white-matter regions from both hemispheres (brainstem, eroded subcortical white matter, corpus callosum anterior, corpus callosum mid_anterior, caudal anterior cingulate cortex, postcentral gyrus, rostral anterior cingulate cortex, ventral diencephalon). To aid with visualization, we show a representation of the brain,^34^ with the regions in the numerator in blue, and the ones in the denominator in red, in Supplementary Fig. 3.

Overall, performance of baselines in Experiment 1 went largely as expected for this preclinical trial scenario. For example, SUVRs with target/numerator regions involving later Braak stages performed poorly. The next worse targets were Braak2 (expected because of off-target binding) and temporoparietal, with mostly SSE above 1000. The best results of literature-inspired biomarkers in terms of SSE were obtained with the mesial temporal as a target (simple average of its regions’ SUV), with an SSE of 206, when using a reference region consisting of the simple average of the eroded subcortical white matter and the cerebellum regions. The next best targets were Braak stages 1 and 3 (SSE of 253 and 256, respectively, using the average SUV), when using a reference region consisting of the average of the eroded subcortical white matter and inferior cerebellum grey matter.

### Experiment 2 (hypothetical 18-month clinical AD trial)

**Figure 3:**
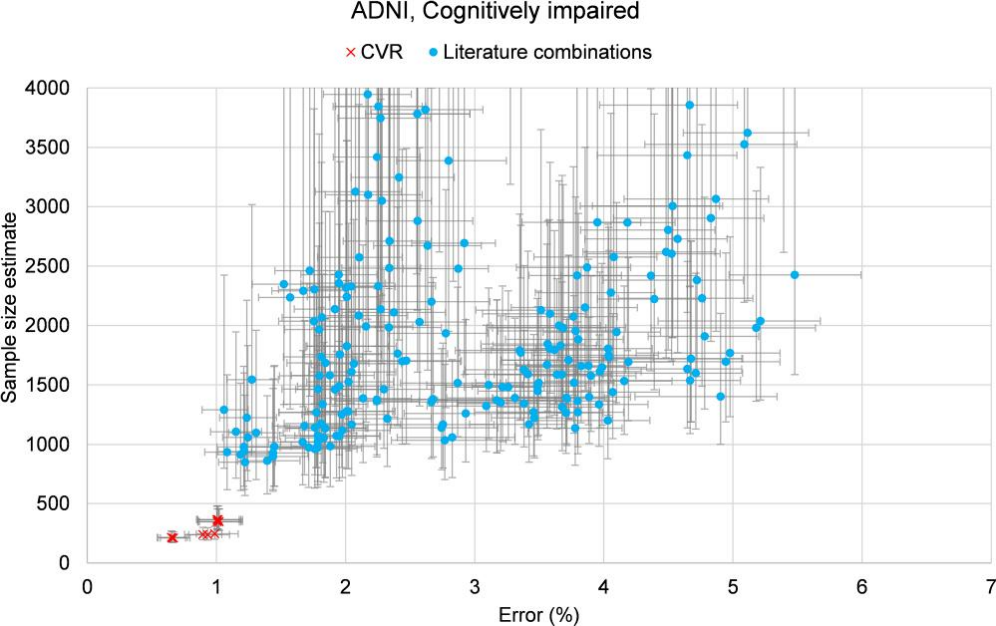
**Biomarker performance for Experiment 2.** The plot shows SSE against percentage error (longitudinal precision) for all biomarkers in Experiment 2, truncated at SSE ≤ 4000. CVR biomarkers (red crosses) showed superior performance to baseline comparators (blue dots), reducing both the SSE and the error. Error bars show 95% confidence intervals from bootstrapping. The SSE shown is the minimum number of individuals that would be needed per branch for a hypothetical clinical trial (CI individuals), designed for 80% power and 20% effect size, for a duration of 1.5 years.

Figure 3 is a plot of SSE against percentage error (longitudinal precision) for all biomarkers in Experiment 2, truncated at SSE ≤ 4000. CVR biomarkers (red crosses) showed superior performance to baseline comparators (blue dots). Error bars show 95% confidence intervals from bootstrapping (see Statistical analysis).

Table 3 highlights selected biomarkers for Experiment 2 (all results are included in Supplementary material File Results.xlsx as a spreadsheet). Namely, specific literature biomarkers, plus the best-performing baseline (biomarker inspired by the literature) and our CVR biomarkers found by the algorithm. Overall, Table 3 shows that, like with Experiment 1 above, one of the CVR biomarkers always outperforms state-of-the-art biomarkers from the literature—for all metrics and, in particular, our primary goal of smaller SSE.

**Table 3:** Results of Experiment 2: biomarker performance for an 18-month clinical trial in an amyloid-positive, CI cohort

Biomarker	SSE	Separation	Repeatability
Meta-temp/composite	1539 (1091, 2333)	4.37 (2.46, 6.49)	4.67 (3.97, 5.06)
DDS/inferior_cerebellum_gm	1031 (739, 1539)	3.48 (1.57, 5.7)	3.12 (2.63, 3.69)
SUV Braak5/(ewm + cerebellum)	852 (569, 1415)	4.14 (2.3, 6.35)	1.22 (1.02, 1.46)
CVR-SUV-L	348 (275, 454)	5.09 (3.17, 7.3)	1.02 (0.86, 1.19)
**CVR-mSUV-L**	**212** (**174, 265)**	**6.3** (**4.38, 8.42)**	**0.66** (**0.55, 0.76)**
CVR-SUV-B	365 (288, 479)	5.15 (3.13, 7.32)	1.01 (0.85, 1.19)
CVR-mSUV-B	238 (195, 297)	5.6 (3.58, 7.6)	0.93 (0.79, 1.1)

Numbers in parentheses correspond to the 95% confidence interval. SSE is assessed with 80% power to detect a 20% effect size with respect to placebo, with two visits separated by 18 months. In bold, the best result per metric. Composite = average PET SUV of whole cerebellum, brainstem and eroded subcortical white matter. Inferior_cerebellum_gm = inferior cerebellum grey matter. Our biomarkers have the prefix ‘CVR’, short for composite value ratio, with mSUV for mean SUV or SUV for volume-weighted SUV, and a suffix L or B indicating a lateral or bilateral regional analysis.

The best results were obtained with CVR-mSUV-L, which—like the results of Experiment 1—is a ratio of simple-mean SUV combinations with laterality. The numerator included grey-matter regions from both hemispheres (inferior temporal gyrus, insula, medial orbitofrontal cortex, superior frontal gyrus, temporal pole), from the left hemisphere (lingual gyrus, pars opercularis, superior parietal lobule), from the right hemisphere (fusiform, pars orbitalis, pars triangularis, pericalcarine cortex, precuneus, rostral middle frontal gyrus, superior temporal gyrus). The denominator included white- and grey-matter regions from both hemispheres (caudal anterior cingulate cortex, rostral anterior cingulate cortex, brainstem, whole cerebellum), from the left hemisphere (lateral orbitofrontal gyrus, pars orbitalis), from the right hemisphere (lateral occipital sulcus, transverse temporal gyrus).

The second-best SSE in Experiment 2 (about 12% bigger SSE-worse) was obtained by CVR-mSUV-B, as with Experiment 1, which involves bilateral mean SUV. The numerator included the following grey-matter regions from both hemispheres (inferior temporal gyrus, insula, medial orbitofrontal cortex, precentral gyrus, superior frontal gyrus, temporal pole). The denominator included the following white- and grey-matter regions from both hemispheres (brainstem, whole_cerebellum, caudal anterior cingulate cortex, lateral orbitofrontal gyrus, rostral anterior cingulate cortex). To aid with visualization, we show a representation of the brain,^34^ with the regions in the numerator in blue, and the ones in the denominator in red, in Supplementary Fig. 3.

In this experiment as well, performance of baselines went largely as expected for this clinical trial scenario. The earlier Braak stages (1–3) and the subset of their regions that compose the mesial temporal had the worst performance. The best results of literature-inspired biomarkers in terms of SSE were obtained with the Braak5 composite as a target (volume-weighted SUV), with an SSE of 852, when using a reference region consisting of the average of the eroded subcortical white matter and the cerebellum regions. Following this, the next best SSE was 862, achieved by the same Braak5 target regions (mean SUV) and the composite reference region. The next best target was the temporoparietal, using the composite reference region, with an SSE of 962.

### Experiment 3 (hypothetical 18-month clinical AD trial): validation

Table 4 shows the salient results from the discovery set, and their performance in the validation dataset. As mentioned in 2.2 Data, we had to use a subset of intersecting regions of all datasets, hence the differences with Experiment 2. The best result in the validation set was obtained by the CVR-mSUV-B, which was the second-best BioDisCVR configuration in both Experiments 1 and 2. The CVR-mSUV-B numerator consisted in the following grey-matter regions from both hemispheres (inferior temporal gyrus, insula, medial orbitofrontal cortex, precentral gyrus, superior frontal gyrus, temporal pole), while the denominator consisted of the following white- and grey matter regions (brainstem, whole cerebellum, caudal anterior cingulate cortex, lateral orbitofrontal gyrus, rostral anterior cingulate cortex). To aid with visualization, we show a representation of the brain,^34^ with the regions in the numerator in blue, and the ones in the denominator in red, in Supplementary Fig. 3. The second-best result in the validation set was obtained by the adaptive DDS biomarker, although it did not perform as well in the discovery dataset.

**Table 4:** Results of Experiment 3: biomarker performance for an 18-month clinical trial in an amyloid-positive, CI cohort

Biomarker	SSE (discovery)	SSE (validation)	Repeatability (discovery)	Repeatability (validation)
Meta-temp/whole_cerebellum	1722 (1190, 2710)	690 (394, 1509)	4.68 (4.01, 5.07)	2.16 (1.60, 2.84)
DDS/inferior_cerebellum_gm	1031 (739, 1539)	417 (271, 723)	3.12 (2.63, 3.69)	2.08 (1.52, 2.77)
mSUV temporoparietal/ cerebellum	1136 (719, 2056)	755 (419, 1748)	1.84 (1.53, 2.17)	2.20 (1.68, 2.87)
CVR-SUV-L	473 (362, 644)	601 (351, 1261)	1.17 (0.98, 1.38)	0.97 (0.72, 1.26)
CVR-mSUV-L	**326** (**257, 427)**	513 (307, 1027)	**0.60** (**0.51, 0.70)**	**0.63** (**0.47, 0.82)**
CVR-SUV-B	513 (384, 723)	630 (361, 1369)	1.31 (1.1, 1.54)	1.17 (0.88, 1.52)
CVR-mSUV-B	362 (284, 478)	**334** (**217, 579)**	0.85 (0.71, 1.01)	0.70 (0.53, 0.91)

Numbers in parentheses correspond to the 95% confidence interval. SSE is assessed with 80% power to detect a 20% effect size with respect to placebo, with two visits separated by 18 months. In bold, the best result per metric. Composite = average PET SUV of whole cerebellum, brainstem and eroded subcortical white matter. Inferior_cerebellum_gm = inferior cerebellum grey matter. Our biomarkers have the prefix ‘CVR’, short for composite value ratio, with mSUV for mean SUV or SUV for volume-weighted SUV, and a suffix L or B indicating a lateral or bilateral regional analysis.

### Additional experiments

Supplementary Table 1 shows BioDisCVR results when fixing either the numerator (meta-temporal) or the denominator (inferior cerebellum grey matter). It can be observed that allowing BioDisCVR to find a data-driven denominator has a considerable impact in improving both the SSE (by a factor of 3 and 4 for Experiments 1 and 2, respectively, while fixing the reference as the inferior cerebellum grey matter achieves comparable results with literature biomarkers. Full information on regions selected is given in Supplementary File Results.xlsx. Regional ablation analysis is shown in Supplementary Fig. 1, where we re-evaluate a biomarker after removing one region, shows remarkably consistent performance suggesting that no single region dominates the CVR biomarkers, with the exception of the biomarker tested in Experiment 3—validation set, where removing the inferior temporal gyrus reduces the performance considerably (from 362 to 854 SSE).

Interestingly, the error and SSE of CVR_mSUV-B designed for Experiment 1, but applied to Experiment 2 is 0.90 and 652, respectively. The same configuration (CVR_mSUV-B), but trained for Experiment 2, has an error and SSE of 0.85 and 389, respectively, in Experiment 1.

## Discussion

When applied to preclinical (secondary prevention) and clinical (treatment) trial scenarios on Alzheimer’s disease, our data-driven framework BioDisCVR discovered ratio-based tau PET biomarkers that are vastly superior to previous work. As discussed below, this can result in remarkable benefits including time and cost savings when running clinical trials on Alzheimer’s disease.

In a 54-month preclinical trial (Experiment 1), the best BioDisCVR output (mSUV-L) reduced SSE by 82%, decreased repeatability error by 53% and increased group separation by 24% compared to the best result from the literature: the adaptive DDS biomarker.^11^ Compared with the best combination of targets and references inspired by literature, the improvement is still vast, at 62% reduced SSE, 25% less error and about 25 times higher group separation, as the benchmark performed poorly in group separation.

We found similar results in Experiment 2 (18-month clinical trial), where our best biomarker reduced SSE by 79%, decreased repeatability error by 79% and improved group separation by 80%, with respect to the DDS.^11^ As the SSE is proportional to the inverse squared effect size, the CVR biomarkers will remain superior no matter which effect size is chosen.

In Experiment 3 (18-month clinical trial), results showed that the variant of CVR that had the best generalization in the validation dataset was the mSUV-B (mean SUV, bilateral analysis), with a 20% reduction of SSE and 65% reduction of error with respect to the best biomarker from the literature (DDS^11^). A key difference between DDS and CVR is that in our configuration, CVR is a ratio of fixed regions, whereas DDS is adaptive, and different individuals will have different target regions. Notably, this CVR vastly outperformed DDS in the discovery set (68% smaller SSE, 80% smaller error).

The mSUV-B CVR for Experiments 1 and 2 had similarities in the numerator (entorhinal cortex, amygdala, precuneus, superior frontal gyrus, transverse temporal gyrus) and the denominator (brainstem, eroded subcortical white matter, caudal anterior cingulate, rostral anterior cingulate), but specialized in different regions to adapt to the stage of the disease: cortical regions that were stable to use in the denominator for earlier stages (e.g. cuneus) disappeared from the denominator and appeared in the numerator in Experiment 2, as they show changes later in the disease process.^29^ We advise caution in drawing conclusions comparing the few CVR biomarkers among each other, though, since multiple configurations show similar performance (see ablation analysis in Supplementary Fig. 1).

Our framework demonstrates remarkable flexibility, which we exploited in multiple ways. For example, in supplementary experiments, we incorporated priors and constraints to enhance interpretability and bolster confidence in the main findings. Allowing for hemispheric laterality generally yielded CVR biomarkers with slightly improved SSE (5–30% smaller), which seems to concur with a previous finding of laterality of tau pathology accumulation in Alzheimer’s disease.^35^ In addition to the volume-weighted composite SUV that is used in previous SUVR studies on statistical ROIs,^12-16^ we considered a regional mean SUV, remarkably finding that the regional mean produced CVR biomarkers with better repeatability error (7–48% smaller) and SSE (35–60% smaller). The effect of composition was not significant when performing a Wilcoxon rank-sum test in the literature-inspired biomarkers (*W* = 19944, *P*-value = 0.7079, filtering out biomarkers that had above 3000 SSE). Braak stage SUVR biomarkers (using the composite reference region) produced results comparable to the best biomarkers from the literature, but still worse than CVR (see Supplementary File Results.xlsx, tab Exp1_and_2). As could be expected, the earliest Braak Stage 1 was favoured for Experiment 1 (preclinical trial), and the later Braak Stage 5 was favoured for Experiment 2 (clinical trial). Fixing the numerator as the meta-temporal region (and finding a data-driven denominator) improved results compared to using the meta-temp/composite biomarker.^9^ Likewise, fixing the denominator as the inferior cerebellum grey matter yielded improved results compared to the literature biomarkers, providing further support for our approach of moving beyond the traditional reference regions used in SUVR analyses. Exploring alternative optimizers as a search algorithm (box-constrained BFGS or Nelder-Mead) did not achieve results on par with the ones obtained by the genetic algorithm (results not shown), but other optimization approaches could be explored in the future. Different random initializations generated similarly performing biomarkers, with a low coefficient of variation (0.043) in fitness for Experiment 2 (clinical), but seemed to stop at a local minima for one of the random seeds in Experiment 1 (preclinical), with a coefficient of variation of 0.17. As observed in the regional ablation analysis (Supplementary Fig. 1), where different biomarkers exhibited similar performances, at the final computed generation of the genetic algorithm, the top 5 biomarkers exhibited comparable performance metrics, with a fitness coefficient of variation below 10% for Experiment 1 (preclinical) and below 5% for Experiment 2 (clinical), (Supplementary File Results.xlsx). The framework’s adaptability extends beyond the two experimental scenarios (54- and 18-month clinical trials) considered in our experiments—it could have easily been employed to identify a biomarker that performs well in both scenarios, thereby providing a unified solution.

Our experiments produced biomarker ratios that included known regions commonly associated with Alzheimer’s disease. The numerator often included regions commonly used as targets in SUVR studies of Alzheimer’s disease progression, e.g. entorhinal cortex, fusiform gyrus, inferior temporal gyrus, middle temporal gyrus. The denominator often included regions used as SUVR references, e.g. eroded subcortical white matter, whole cerebellum or cerebellum cortex, brainstem. The fact that our CVR (mean SUV, bilateral) designed for a trial works on the other (better than the specific two biomarkers from the literature) validates, in unseen data, that they are targeting disease-specific regions. Furthermore, both the 95% confidence intervals and the ablation study on influential regions (Supplementary Fig. 1) suggest a robust fit in the available data, free of questionable influential regions. Additional metrics or priors to guide the algorithm could be used to select a smaller set of good-performing biomarkers. Results indicate that using a curated biomarker per clinical trial target group can better characterize relevant disease progression, in line with the recent Revised Criteria for Diagnosis and Staging of Alzheimer’s Disease,^6^ reflecting that different regions are affected at different stages of the disease.

The fast execution of the framework BioDisCVR, together with the usage of readily processed scans (thus avoiding the need to re-run preprocessing methods) makes this approach computationally cheap, positively addressing an increasingly concerning environmental impact of machine learning.^36^

We anticipate that data-driven methods such as BioDisCVR could revolutionize the drug development pipeline. The dramatic reduction in SSE produced here has multiple benefits. First, a smaller trial reduces the number of individuals exposed to increased risk of side effects such as ARIA.^37-40^ Second, trials can take considerably less time, e.g. the A4 Study screening time could have been reduced from 44 months^41^ to under 12 months, considering a linear relationship between recruitment time and sample size.^42^ Third, the cost savings could be immense, e.g. for a cost per individual of $40k,^43^ the possible savings per trial using CVR-mSUV-B (as opposed to the best published biomarker) could be [2 arms × (1031–238)×40 000] about $65 million for CI/18-months, or [2 arms × (438–115)×40 000] about $25 million for CU/54-months. In the event that other factors influence the minimum sample size needed and cannot be reduced, using CVR would allow a more precise detection threshold of disease progression. The superior longitudinal precision of our data-driven biomarkers makes them candidates for secondary outcomes or endpoints.

We highlight opportunities for future work. First, it is important to explore further validation of tau PET ratio-based biomarkers across cohorts, ethnicities, etc. (work in progress in our team). Second, our framework can be applied across other PET tracers and beyond PET, e.g. to discover new and improved ratio-based blood biomarkers.^5,6^ Indeed, the small run-time needed for computation facilitates extensive exploration in any data modality and even across modalities, including fluid biomarkers and MRI (less expensive than PET and non-invasive). Not only this, but data acquisition cost could be included in the fitness function to discover a cost-effective ratio-based multimodal biomarker—potentially benefiting low- and middle-income countries, in particular. Third, future work could consider deriving a theoretical guarantee that a given search algorithm will find the global optimum biomarker, but in practice we found that multiple runs with random seeds produced very similar results (see ablation analysis in Supplementary Fig. 1 and top-n biomarkers in Supplementary File Results.xlsx).

## Conclusion

Clinical trials of anti-amyloid and anti-tau therapies in Alzheimer’s disease need a precise and accurate biomarker for assessing disease modification (pathology clearance). We have introduced and deployed a data-driven framework for discovering such biomarkers, with experimental results showing state-of-the-art longitudinal precision, and drastically reduced sample size estimates required for modern clinical trials in symptomatic and preclinical Alzheimer’s disease. Our results suggest that considerable time, money and participant suffering could be saved by incorporating our biomarker framework into the design of future preclinical and clinical trials.

## Supplementary Material

fcae438_Supplementary_Data

## Data Availability

Data used in this work (discovery dataset) can be found in the Alzheimer’s Disease Neuroimaging Initiative (ADNI) database (*adni.loni.usc.edu*). The cohorts used for validation (ADRD and MCSA from Mayo Clinic) can be requested at *mayo.edu* website. The framework code for the discovery of biomarkers presented in this work is provided in R and is available as a Supplementary file (see the online version of this paper at Brain Communications).
