# Supplementary material for: A data-driven framework for biomarker discovery applied to optimizing modern clinical and preclinical trials on Alzheimer’s disease
Source: Brain Commun. 2024 Dec 9;6(6):fcae438. doi: 10.1093/braincomms/fcae438 (PMC11632366; doi:10.1093/braincomms/fcae438)
Supplement: fcae438_Supplementary_Data [file fcae438_supplementary_data.zip › Supplementary_material.docx]

# Supplementary material

**Supplementary Table 1**: **BioDisCVR fixing either the numerator or denominator as known literature ROIs.** We have different designs here: we fix the numerator as the meta-temporal, and find a fitting denominator (CVR_r(ft_meta)). We do the same, but starting by fixing the denominator as the inferior cerebellum grey matter (igm). This configuration of BioDisCVR, considers bilateral regions (using both hemispheres jointly) and takes the mean standardised uptake values (SUV) of the regions. The two first rows show biomarkers from the literature^1,2^, while the third row for each sub-table shows the best-performing biomarker out of 220 literature-inspired combinations of composite targets and references. We can observe the big impact of allowing our algorithm to find an appropriate data-driven composite to be used as a reference (denominator). Abbreviations: composite = average of whole cerebellum, brainstem and eroded subcortical white matter; DDS = adaptive individualised biomarker by Leuzy et al.^2^; ewm = eroded subcortical white matter; igm = inferior cerebellum grey matter; SSE = sample size estimate (80% power, 20% effect size, 4.5 years for Experiment 1, and 1.5 years for Experiment 2).

| Experiment 1 (cognitively unimpaired), with fixed numerator or denominator | | | |  |
| --- | --- | --- | --- | --- |
| **Numerator** | **Denominator** | **SSE** | **Separation** | **Repeatability** |
| meta-temporal^3^ | composite^4^ | 626 (353, 1401) | 4.37 (2.46, 6.49) | 1.06 (0.82, 1.31) |
| DDS^2^ | igm | 438 (259, 895) | 3.48 (1.57, 5.7) | 1.66 (1.32, 2.06) |
| mesial temporal (mean) | (ewm+cerebellum) | 206 (143, 325) | 0.17 (-1.84, 2.09) | 1.03 (0.82, 1.3) |
| meta-temporal^3^ | CVR_r(ft_meta) | 207 (143, 329) | 2.23 (0.25, 4.18) | 1.16 (0.91, 1.43) |
| CVR_t(igm) | igm | 321 (201, 591) | 2.82 (0.81, 4.78) | 1.46 (1.13, 1.79) |
|  |  |  |  |  |
| Experiment 2 (cognitively impaired), with fixed numerator or denominator | | | |  |
| **Numerator** | **Denominator** | **SSE** | **Separation** | **Repeatability** |
| meta-temporal^3^ | composite^4^ | 1539 (1091, 2333) | 4.37 (2.46, 6.49) | 4.67 (3.97, 5.06) |
| DDS^2^ | igm | 1031 (739, 1539) | 3.48 (1.57, 5.7) | 3.12 (2.63, 3.69) |
| Braak5 | (ewm+cerebellum) | 852 (569, 1415) | 4.14 (2.3, 6.35) | 1.22 (1.02, 1.46) |
| meta-temporal^3^ | CVR_r(ft_meta) | 372 (290, 495) | 3.8 (1.85, 5.87) | 1.55 (1.32, 1.82) |
| CVR_t(igm) | igm | 1052 (722, 1672) | 4.49 (2.54, 6.64) | 2.58 (2.16, 3.03) |


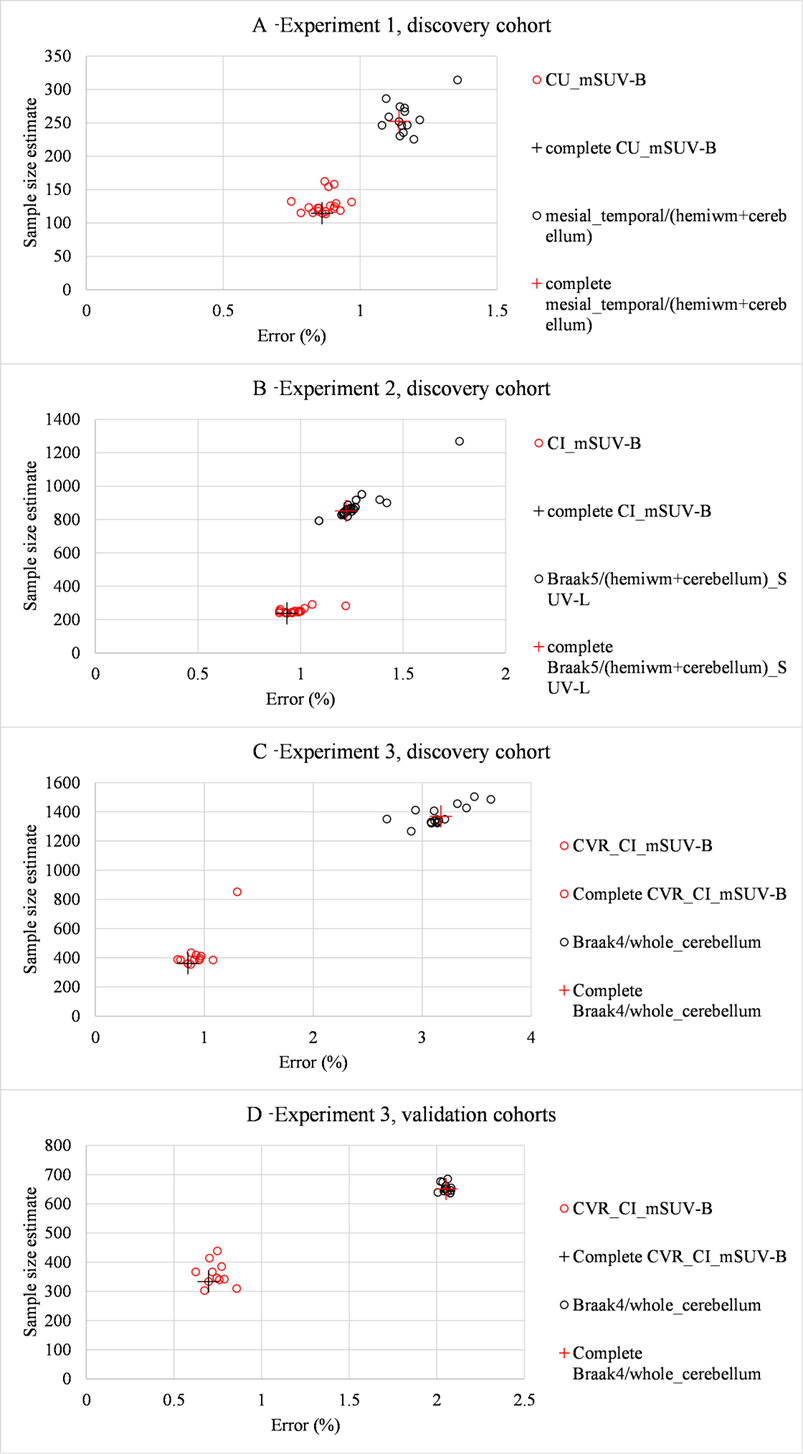


**Supplementary Figure 1: Repeatability (error) and SSE performance of selected biomarkers**, removing one region at a time, for cognitively unimpaired (A) and cognitively impaired (B). Panels C and D show the performance of the discovery and validation sets, respectively, using the brain regions that were available in both datasets. The crosses represent the performance of the full, original biomarker. The vast worsening of SSE meta-temp/composite is due to removing the eroded subcortical white matter region from the denominator, which is also present in both CVR biomarkers. A similar observation is seen when the inferior temporal gyrus is removed from the numerator in (C) CVR_mSUV-B, highlighting its importance in the biomarker. Abbreviations: CVR = composite value ratio, as in “the ratio of two composite regions”. SUV = standardised uptake value, the signal measured in a positron emission tomography scan over a volume. The prefix “m” in SUV indicates that it is the mean SUV over a number of regions; otherwise, it is the volume-weighted SUV. The suffix -B after SUV indicates that the analysis was bilateral, as in “considering regions in both hemispheres”, as opposed to -L, which would indicate laterality.

**
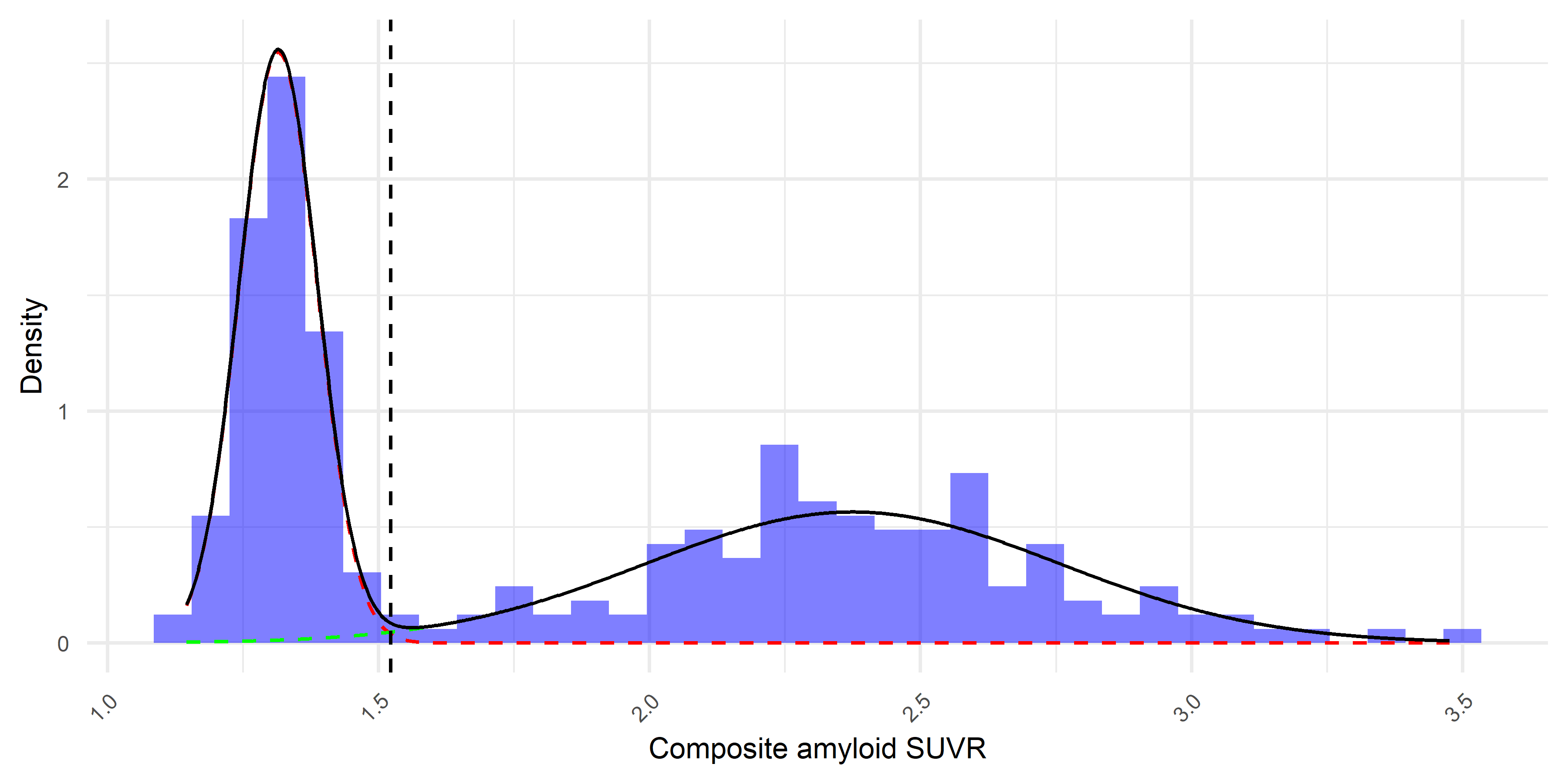
**

**Supplementary Figure 2: Amyloid distribution of the validation set.** The cutoff for amyloid positivity was defined as the intersection of the two Gaussians that lies between their means. SUVR is the standardised uptake value ratio. The reference was the inferior cerebellum grey matter, and the target was the cortical summary region from Lee J, Murphy A, Ward T, Harrison T, Landau S, Jagust W.: Amyloid PET Processing Methods, published online 2023 in the ADNI database^5^.


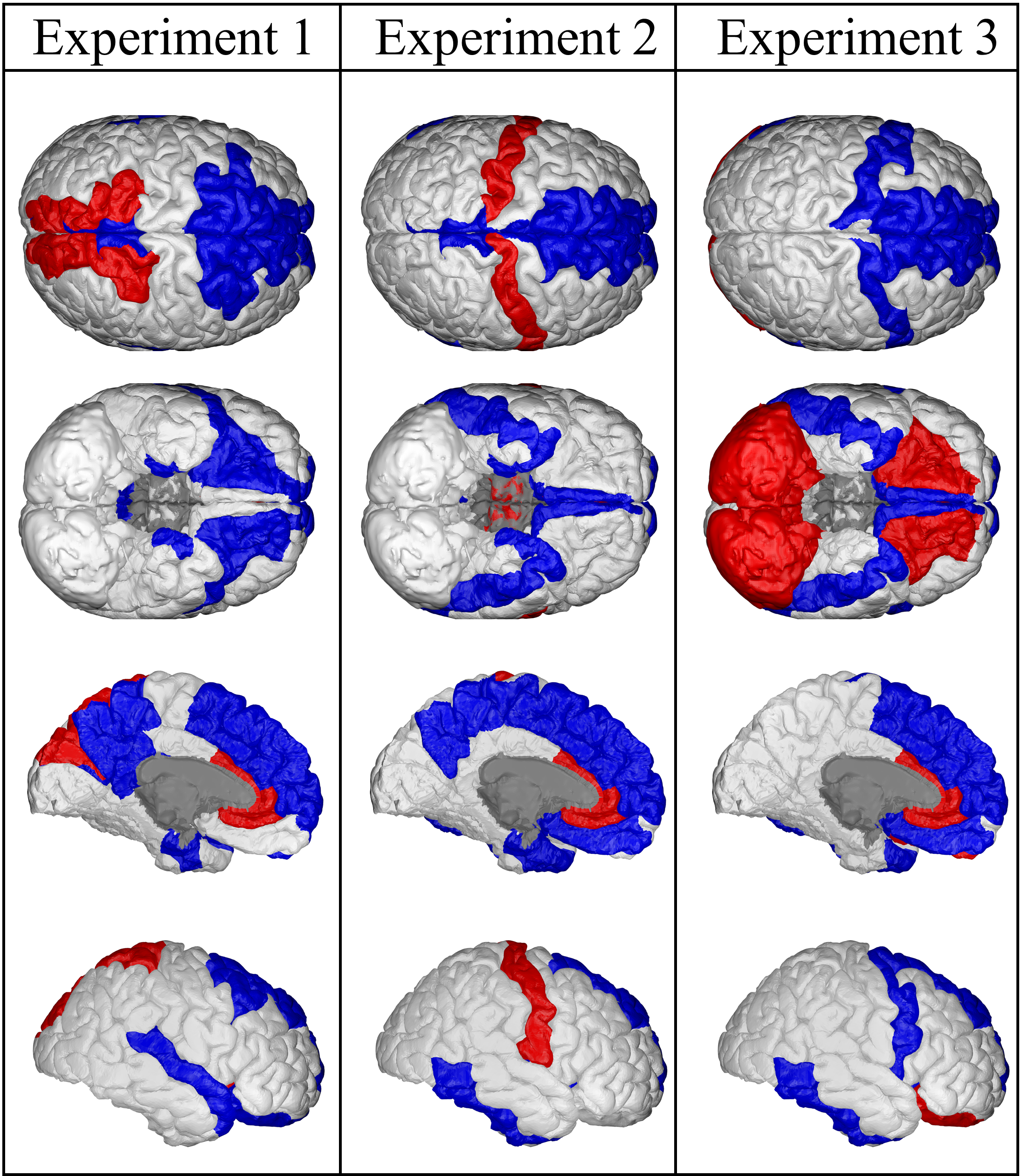


**Supplementary Figure 3: Visualization^6^ of numerator (blue) and denominator (red) regions for our biomarker CVR-mSUV-B, for Experiments 1, 2 and 3.** CVR stands for “composite value ratio”. In this case, the composition is the mean of the regions. The suffix -B indicates that the design considered bilateral regions (joint left- and right-hemisphere).

**References**

1. Schwarz CG, Therneau TM, Weigand SD, et al. Selecting software pipelines for change in flortaucipir SUVR: Balancing repeatability and group separation. *NeuroImage*. 2021;238:118259. doi:10.1016/j.neuroimage.2021.118259

2. Leuzy A, Binette AP, Vogel JW, et al. Comparison of Group-Level and Individualized Brain Regions for Measuring Change in Longitudinal Tau Positron Emission Tomography in Alzheimer Disease. *JAMA Neurol*. 2023;80(6):614. doi:10.1001/jamaneurol.2023.1067

3. Jack CR, Wiste HJ, Weigand SD, et al. Age-specific and sex-specific prevalence of cerebral β-amyloidosis, tauopathy, and neurodegeneration in cognitively unimpaired individuals aged 50–95 years: a cross-sectional study. *The Lancet Neurology*. 2017;16(6):435-444. doi:10.1016/S1474-4422(17)30077-7

4. Landau SM, Fero A, Baker SL, et al. Measurement of Longitudinal β-Amyloid Change with 18F-Florbetapir PET and Standardized Uptake Value Ratios. *Journal of Nuclear Medicine*. 2015;56(4):567-574. doi:10.2967/jnumed.114.148981

5. Lee J, Murphy A, Ward T, Harrison T, Landau S, Jagust W. ADNI amyloid PET processing methods, 2023.

6. Marinescu RV, Eshaghi A, Alexander DC, Golland P. BrainPainter: A Software for the Visualisation of Brain Structures, Biomarkers and Associated Pathological Processes. In: Zhu D, Yan J, Huang H, et al., eds. *Multimodal Brain Image Analysis and Mathematical Foundations of Computational Anatomy*. Springer International Publishing; 2019:112-120. doi:10.1007/978-3-030-33226-6_13
